# Social Norms of Cooperation in Small-Scale Societies

**DOI:** 10.1371/journal.pcbi.1004709

**Published:** 2016-01-25

**Authors:** Fernando P. Santos, Francisco C. Santos, Jorge M. Pacheco

**Affiliations:** 1 INESC-ID and Instituto Superior Técnico, Universidade de Lisboa, IST-Tagusparque, Porto Salvo, Portugal; 2 ATP-Group, Lisboa, Portugal; 3 Centro de Biologia Molecular e Ambiental, Universidade do Minho, Braga, Portugal; 4 Departamento de Matemática e Aplicações, Universidade do Minho, Braga, Portugal; Monash University, AUSTRALIA

## Abstract

Indirect reciprocity, besides providing a convenient framework to address the evolution of moral systems, offers a simple and plausible explanation for the prevalence of cooperation among unrelated individuals. By helping someone, an individual may increase her/his reputation, which may change the pre-disposition of others to help her/him in the future. This, however, depends on what is reckoned as a good or a bad action, i.e., on the adopted social norm responsible for raising or damaging a reputation. In particular, it remains an open question which social norms are able to foster cooperation in small-scale societies, while enduring the wide plethora of stochastic affects inherent to finite populations. Here we address this problem by studying the stochastic dynamics of cooperation under distinct social norms, showing that the leading norms capable of promoting cooperation depend on the community size. However, only a single norm systematically leads to the highest cooperative standards in small communities. That simple norm dictates that only whoever cooperates with good individuals, and defects against bad ones, deserves a good reputation, a pattern that proves robust to errors, mutations and variations in the intensity of selection.

## Author Summary

The prevalence of cooperation among human societies is a puzzle that has caught the eye of researchers from multiple fields. Why is that people are selfless and often incur costs to aid others? Reputations are intimately linked with the answer to this question, and so are the social norms that dictate what is reckoned as a good or a bad action. Here we present a mathematical framework to analyze the relationship between different social norms and the sustainability of cooperation, in populations of arbitrary sizes. Indeed, it is known that cooperation, norms, reciprocity and the art of managing reputations, are features that go along with humans from their pre-historic existence in small-scale societies to the contemporary times, when technology supports the interaction with a large number of people. We show that population size is relevant when evaluating the merits of each social norm and conclude that there is a social norm especially effective in leveraging cooperation in small populations. That simple norm dictates that only whoever cooperates with good individuals, and defects against bad ones, deserves a good reputation.

## Introduction

Indirect Reciprocity (**IR**), which involves reputation and status [1], constitutes, perhaps, the most elaborated and cognitively demanding mechanism of cooperation discovered so-far [2]. Unlike other mechanisms of cooperation, **IR** has been heralded as providing the biological basis of our morality [1]. Whereas under direct reciprocity one expects to receive help from someone we have helped before, under **IR** one expects a return, not from someone we helped, but from someone else: In this sense, helping the “right” individuals may increase the chance of being helped by someone else at a later stage.

Seminal work carried out since the mid eighties [1–35] has shown how **IR** can lead to the emergence and sustainability of cooperation. Most theoretical models employed to date (for exceptions, see [10,26]) have considered infinite populations. In this context, the work of Ohtsuki and Iwasa [13] became an inspiring and influential framework on top of which many other models were built, and led to the identification of the so-called leading eight social norms of cooperation [13–15].

But what about small-scale societies, e.g., Hunter-Gatherers where reputation is paramount [36,37]? Indeed, and despite other forms of reciprocity or kinship relations that may also play a co-evolutionary role, reputations easily diffuse in small communities and influence individuals’ choices. In this context, it remains an open question which norms are able to promote cooperation in small societies. Here we shall investigate to which extent norms found to promote cooperation in large populations will remain effective in small societies, and also to which extent the capacity of a social norm to foster cooperation depends on the community size.

In small populations, stochastic finite size effects are not only important, but may even render analyses based on concepts originating from infinite populations misleading. In the context of direct reciprocity, for instance, it was shown that individuals in finite populations select reciprocation, while defection is selected in infinite populations [38]. In general, it is also well-known that strict Nash Equilibria and Evolutionary Stable Strategies may not prevail in finite populations [39,40,41,42]. In this paper we address this problem by studying the stochastic dynamics of different strategies (also called action or behavioral rules) when reputation assignment is governed by second order social norms (defined below).

Consider a finite population comprised of *Z* individuals who may opt to help one another (that is, to Cooperate, ***C***) or not (to Defect, ***D***). Random pairs of individuals are chosen and play the donation game, one being the potential provider of help (donor) to the other (recipient). The donor may cooperate and help the recipient at a cost *c* to herself/himself, conferring a benefit *b* to the recipient (with *b* > *c*). The donor may also decide not to help, in which case no one pays any costs nor distributes any benefits. In line with previous work, this donation game characterizes the interactions between pairs of individuals in the population. We further assume that individuals have a public reputation that can only have 2 attributes: *Good* (***G***) or *Bad* (***B***). It is worth pointing out that, to begin with, ***G*** and ***B*** reputations are mere labels with no *a-priori* meaning. Their significance will eventually emerge in association with individual behavior in connection with the donation game. Indeed, it is the structure of the donation game, in which help implies engaging in a costly action to confer a higher benefit to someone else, that ultimately assigns a meaning to the reputation labels.

Decision is an individual attribute, encoded in a behavioral rule specified by the duple *p* = (*p*_*G*_,*p*_*B*_) that defines the probability of an individual to opt for ***C*** when facing a ***G*** and a ***B*** opponent, respectively. The reputation of each individual is public and (errors apart, see Methods) is attributed by a bystander who witnesses a pairwise interaction; in doing so, she/he identifies the action (***C*** or ***D***) of the donor, as well as the reputation (***B*** or ***G***) of the recipient, based on which she/he attributes a new reputation to the donor. To perform this task, the bystander uses a social norm, that is, a rule that converts the combined information stemming from the action of the donor and the reputation of the recipient into a new reputation for the donor. Social norms encoding this type of information are classified as second-order norms [13–15,26]. In this hierarchy, first-order norms convert the action of the donor into a new reputation for her/him, whereas third-order norms use, besides the information used in second-order norms, the reputation of the donor at the time of engaging in the donation game. Likewise, the complexity of behavioral rules varies concomitantly. In the space of second order norms we shall consider here, the duple *p* suffices to unambiguously define a strategy, leading to the following 4 possible strategies: *unconditional Defection* (*AllD*,*p* = (0,0)), *unconditional Cooperation* (*AllC*,*p* = (1,1)), *Discriminator strategy* (*Disc*,*p* = (1,0)), that is, cooperate with those in *good* standing, and defect otherwise), and *paradoxical Discriminator strategy* (*pDisc*,*p* = (0,1), the opposite of *Disc*).

This simplified societal structure has been very influential in studying the evolution of cooperation under indirect reciprocity [4,7,9,10,13–15,17–19,22,23,25–27]. Unlike previous analytical studies, however, we shall investigate the evolutionary dynamics of small-scale societies by means of stochastic birth-death processes, monitoring explicitly to which extent a social norm fosters cooperation.

Let us assume that all individuals start with the same reputation (say, ***G***), and that some of them (*k ≤ Z*) adopt the behavioral rule *p* while the rest of the population (*Z-k*) adopts another behavioral rule *p’*. By interacting with each other, it may happen that individual reputations change in time. If no one changes their behavioral rule, there will be a characteristic time after which the distribution of reputations in the population will stabilize. This stable distribution can be determined by computing the limiting distribution of the 2-dimensional Markov chain described in the Methods section. Given a (stationary) distribution of reputations, we can compute the fitness of an individual using behavioral rule *p* (*p’*) by determining the average payoff of such an individual in the population.

Knowledge of the fitness of each type of individual in the population allows us now to study the evolution of behavioral rules in the population. To this end we define a stochastic birth-death process. Analytically, we shall restrict the number of behavioral rules present in the population, at any time, to be at most two. In other words, we assume that no new behavior rule appears in the population before one of the 2 existing (*p* and *p’*) rules goes extinct. Such a Small Mutation Approximation (**SMA**) [43], which has been employed in the past with great success [40–45]—albeit not in the context of **IR**—allows us to compute, for a population under a given social norm *i)* the stationary distribution of behavioral rules and, from it, *ii)* the *cooperation index* (η, a real number between 0 and 1, defined in Methods) of that population, measuring the average fraction of donations observed in a community evolving under a given social norm. Computer simulations, in which all behavior rules are allowed to co-evolve, allow us to show that the intuitive analytical results extracted from the **SMA** do actually remain valid in a surprisingly wide parameter range (see S1 Text).

## Results

**Fig 1:**
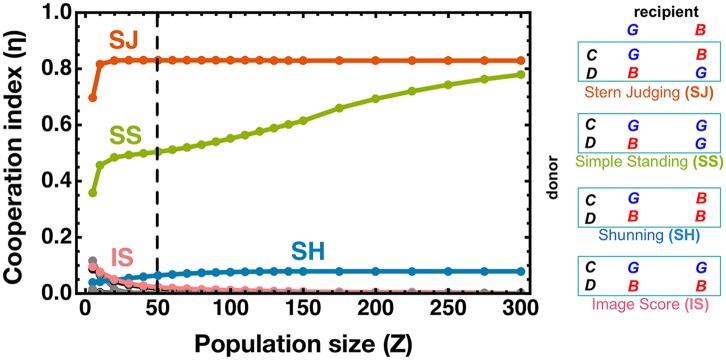
Cooperation index η under the influence of different social norms. Analytical results provided by the **SMA**. **SJ** consistently leads to the highest values of the η for all community sizes. **SS**—the other social norm belonging to the leading-two set [15]—supports significant levels of cooperation, yet always below the index values attained by **SJ**. **SH** looses due to its prohibitive strictness, which may often label discretionary individuals as *bad*, discouraging cooperation (see main text for details). **IS** performs generally worse than the other 3, except for very small population sizes. The results regarding other norms are colored with either gray or black; most lead to index values close to 0. The table explicitly defines each of the 4 dominant social norms in terms of the action (***C*,*D***) of the donor and the reputation (***B*,*G***) of the recipient. Other parameters are (see Methods for explicit definition): *b = 5*, *c = 1*,*α* = *χ* = 0.01,*ε* = 0.08,*β* = 1. The vertical dashed line indicates the population size (*Z* = 50) at which the fixation probabilities and the fraction of time spent in each monomorphic configuration were calculated and discussed later in the text.

In Fig 1 we calculate analytically the cooperation index (η) for different social norms as a function of (small) population size.

Out of a total of 16 second order social norms [15], only 10 are truly distinct, and of these, four have been given special attention: ***Stern-judging*** [14,26,33] (**SJ**, also known as *Kandori*, which assigns a *good* reputation to a donor that helps a *good* recipient or refuses help to a *bad* one, assigning a *bad* reputation in the other cases); ***Simple***-***Standing*** (**SS**) [5], similar to **SJ**, but more “benevolent” by assigning a *good* reputation to any donor that cooperates; ***Shunning*** (**SH**) [6,18,22], similar to **SJ** but less “benevolent”, by assigning a *bad* reputation to any donor that defects; and ***Image Score*** [12,21] (**IS**, a first order norm) where all that matters is the action of the donor, who acquires a *good* reputation if playing ***C*** and a *bad* reputation if playing ***D***.

The results in Fig 1 show that **SJ** is able to foster the highest values of the cooperation index η, independently of the (finite and small) population size. Large-scale agent-based computer simulations confirm these results (see S1 Text).

**Fig 2:**
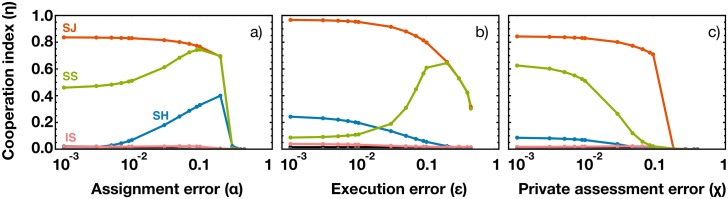
Effect of errors in the cooperation index η provided by different social norms. a) **Assignment error (** *α* **):** We allow for mistakes to happen when a bystander attributes a new public reputation. This kind of error leverages the η in both **SS** and **SH**; b) **Execution error (** *ε* **)**: It prevents an intended donation act to occur. This error acts to increase the η of **SS** and to decrease that of **SH**; c) **Private assessment error (** *χ* **)**: This error leads potential donors to perceive the wrong reputation of the opponent and may affect both the action of a donor or the assignment of a new reputation by a bystander. Private assessment error damages the η of all social norms. In all cases, **SJ** proves robust to noise, remaining the social norm that leads to the highest η. The results regarding the remaining social norms are colored in either gray or black; mostly, these exhibit a zero value for η. The impact of each error is, in general, enhanced for large population sizes. In particular, it can be shown that, for large populations, under low execution or assignment errors, **SJ** emerges as the only norm that promotes cooperation (see S1 Text). Other parameters, when not explicitly varied, are (see Methods for explicit definition): *b = 5*, *c = 1*,*α* = *χ* = 0.01,*ε* = 0.08,*β* = 1,*Z* = 60.

Despite leading to cooperation index values systematically lower than **SJ** in small-scale societies, **SS** is capable of providing significant levels of cooperation. The fact that **SS** is more benevolent than **SJ** towards unconditional cooperators prevents it from sustaining levels of cooperation comparable to **SJ** in small-scale societies. Conversely, **SH** harms cooperation (by being too strict compared to **SJ**) due to the abusive widespread assignment of *bad* labels. The right balance of **SJ**, in turn, proves robust to variations in population size and different error rates, as shown in Fig 2, where the robustness with respect to errors is investigated for each of the four social norms explicitly defined in Fig 1. As also shown in Fig 1, for large populations, the levels of cooperation obtained under **SS** smoothly converge to the levels obtained with **SJ**, confirming these two social norms as the *leading-two* in promoting cooperation [15].

Fig 2 allows to further capture the robustness of each social norm in the presence of noise. We consider errors of assignment, of execution and of private assessment.

The disadvantages of having a norm that is more (**SS**) or less (**SH**) benevolent than **SJ** are highlighted by the impact that each kind of error has on it. **SS** benefits from assignment and execution errors. It happens because those specific errors allow to disambiguate between an unconditional and a conditional cooperator. For example, in a population governed by **SS** and solely composed by *AllC* and *Disc*, everyone would be regarded as ***G***. Mistakenly failing *i*) to donate (execution error) or *ii*) to assign a *good* reputation (assignment error), leads to an increase of ***B*** individuals, providing an advantage to *Disc* individuals. On the contrary, the lack of benevolence of **SH** is alleviated by assignment errors, as ***G*** individuals will now increase (by mistake). Execution errors, in turn, do not promote cooperation under **SH**, as they act to further increase the number of ***B*** individuals (specially in populations dominated by *Disc*), or to explicitly decrease the number of donations.

**Fig 3:**
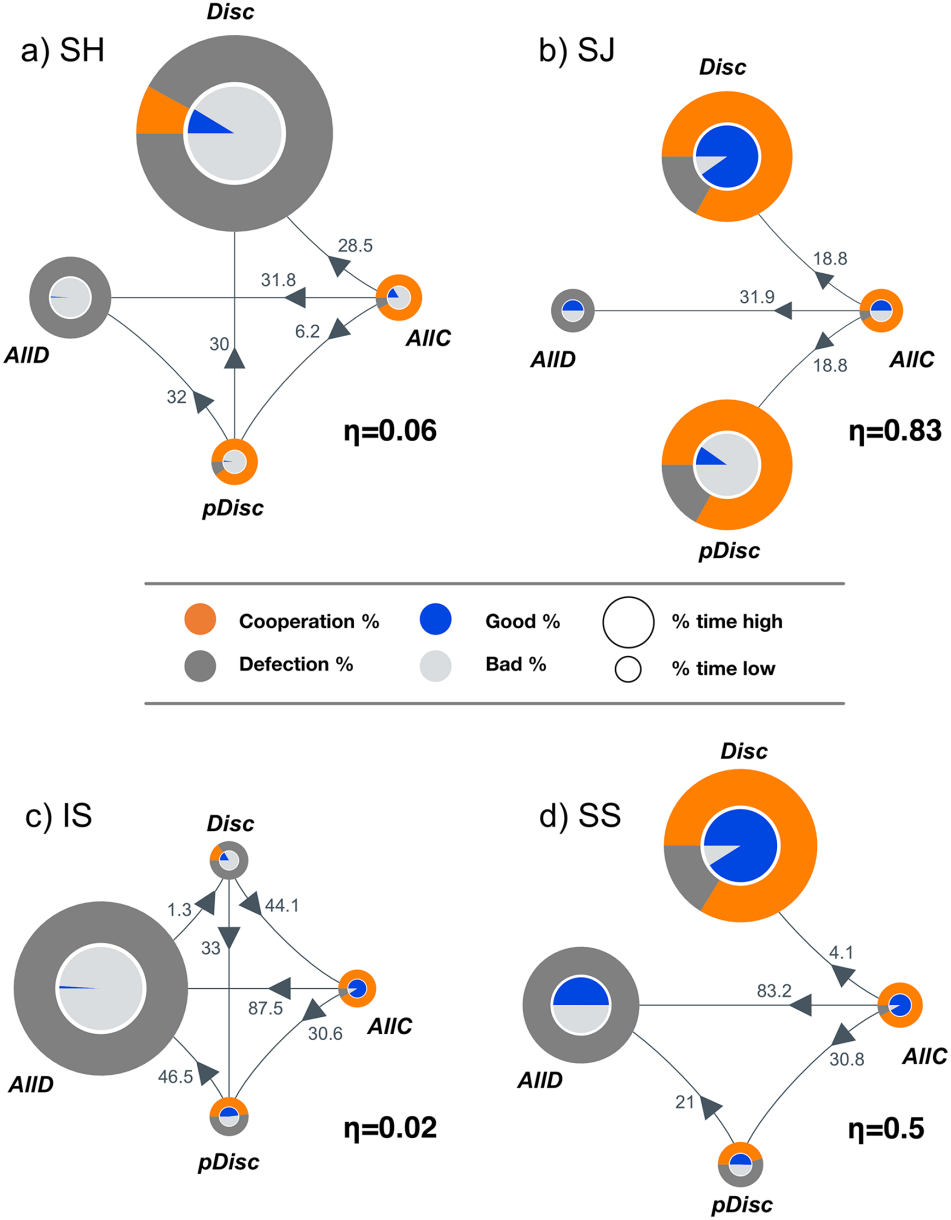
Evolutionary dynamics under each norm. In each graph, each vertex represents one of all 4 possible monomorphic states; each drawn edge represents a transition between strategies occurring above neutral drift (numbers provided are relative to neutral fixation probability 1/*Z*). The radius of each node is proportional to the prevalence of the respective strategy in time. For each state, we show the fraction of cooperative/defective acts by orange/dark-gray pie charts. Similarly, blue and light gray pies display the stationary fraction of ***G*** and ***B*** reputations (see Table A in S1 Text for numerical values). Panel **(a)** indicates that **SH** stabilizes *Disc*, yet with a majority being assigned a ***B*** reputation (light gray color) thus leading to low levels of the *cooperation index* η. A different scenario emerges in panel **(b)** under **SJ**, where individuals spend most of the time in both *Disc* and *pDisc* configurations; in both cases, most individuals cooperate. The more benevolent strategy **SS**, shown in panel **(d)**, as well as the first order norm **IS**, shown in panel **(c)**, are both unable to prevent the population to spend time in *AllD* monomorphic configurations. Parameters (see Methods for details): *Z* = 50, *b = 5*, *c = 1*, α = χ = 0.01, ε = 0.08, β = 1.

While Figs 1 and 2 provide aggregate information regarding the performance of each social norm, they do not reveal the interplay between strategies that is on the basis of the *cooperation indexes* observed. Such an interplay is detailed in Fig 3, where we resort to directed graphs in which each vertex corresponds to one of the four possible monomorphic states and respective strategies: *AllC*, *AllD*, *pDisc* and *Disc*. The radius of each vertex corresponds to the prevalence of each strategy in time, whereas orange/dark-gray pies represent the level of cooperation/defection, while blue/light-gray pies display the stationary fraction of ***G*** and ***B*** reputations at each monomorphic state. Arrows represent the fixation probabilities of one individual (with a strategy located at the vertex of origin of the arrow) in a population of individuals (with a strategy located at the vertex at the end of the arrow).

The values, computed analytically in the **SMA**, are only shown whenever the fixation probability is larger than neutral fixation, given by 1/*Z*, with values reported relative to the neutral fixation value.

Fig 3 shows that, in accord with previous studies [13–15], all the so-called *leading 2* ^*nd*^ *order norms*—**SJ** and **SS**—are able to promote *Disc* to an *evolutionary robust strategy* [46,47], defined as strategies for which no mutant, adopting any other strategy, has a selective advantage. To these leading 2^nd^ order norms, one may also add **SH**, which, despite not being a leading norm, can also make *Disc* an evolutionary robust strategy. This norm, however, is unable to support the *good* standing of *Disc* individuals, a fact that is stressed by execution errors and alleviated by the assignment ones (see Fig 2). **IS**, in turn, is dominated by the *AllD* state, despite the inexistence of any evolutionary robust strategy. Thus, only **SJ** and **SS** are able to combine a high prevalence of an ALL-*Disc* configuration with the incidence of ***G*** reputations in this configuration, efficiently fostering high levels of cooperation. This said, **SS** cannot preclude strong transitions from both *AllC* and *pDisc* into *AllD*, with a significant impact on the overall levels of cooperation (see Fig 1). As a side remark, for large populations, the relative magnitude of these two transitions is reduced in comparison with what is observed in small communities, while the transition from *AllC* to *Disc* is strengthened, leading to the result pictured in Fig 1. The opposite will happen for low execution errors (see S1 Text for details).

Furthermore, **SJ** is the only social norm that profits from the existence of a *pDisc* strategy. Indeed, the population spends roughly half of the time in an ALL-*pDisc* configuration and the other half in an ALL-*Disc* configuration. The symmetry of **SJ**, however, dictates that, in both cases, individuals end up cooperating (apart from errors): cooperate and remain *good* in the ALL-*Disc* configuration, and cooperate and remain *bad* in the ALL-*pDisc* configuration. However, as stated before, the labels ***G*** and ***B*** have no pre-determined meaning in our formulation. What is remarkable with **SJ** is that it is the only social norm that successfully fosters cooperation in the donation game, irrespectively of the labeling adopted. Indeed, *pDisc* is the equivalent to *Disc* when the labels *good* and *bad* are swapped. The specific labeling, in turn, is irrelevant: All that matters—and ultimately defines a moral system—is what is achieved through the donation game.

Finally, but importantly, *i)* the advantages of **SJ** remain valid for different values of errors and selection strength, and, in the presence of errors of execution, *ii)* such advantage is emphasized in small scale societies, as shown already. It is also noteworthy that the analytical results discussed above, obtained in the limit where mutations rarely occur [43], remain valid for a wide range of mutation probabilities, as we show explicitly in the S1 Text via comparison with results from numerical simulations. Additionally, in the S1 Text, we also show that the analytical results remain valid for a wide interval of reputation assignment time-scales, as we abandon the time-scale separation ansatz that sits at the heart of the analytical treatment adopted.

## Discussion

We have investigated the stochastic dynamics of different strategies (behavioral rules) as a function of population size, when reputation assignment is governed by second order social norms. In our model, where the reputation dynamics is also the outcome of a stochastic process, the four social norms among first and second-order norms that lead to a *cooperation index* η higher than 0 are **SJ**, **SS**, **SH** and **IS**. From these, **SJ** clearly stands out for small population sizes, dominating with **SS** for large population sizes, ensuring high values of η that are robust to parameter variations and errors. Interestingly, the fingerprint of both leading norms **SJ** and **SS** is consistent with recent findings showing that toddlers not only positively evaluate those who treat others prosocially [48–51], but also positively evaluate those who behave negatively towards those who have acted antisocially [50]. Moreover, in Ref. [50] it is specifically pointed out that toddlers clearly prefer characters that harm (rather than help) antisocial puppets which fits nicely with the assessment of **SJ**.

On the other hand, the relative importance of **SS** and **SH** depends on the amount and nature of noise. For cases in which individuals often make errors when donating, benevolent social norms are appropriate, and thus, **SS** prevails over **SH**. If execution errors are rare, larger populations and a larger selection pressure (high *β*) allows **SH** to prevail over **SS**, and benevolent social norms become less capable of promoting cooperation. **SS** and **IS**, in turn, benefit from noise, as is the case when populations are very small or when the exploration rate *μ* is large.

Clearly, to assess the effect of a particular social norm regarding the promotion of cooperation in a finite population, it is not enough to require the evolutionary stability or robustness of the discriminating strategy (*Disc*), as addressed in previous works on **IR** [9,15]. When population sizes grow from 5 to 130, a range that includes typical community sizes of hunter-gatherer societies, and in which one expects stochastic effects to play a sizable role, we find that, under **SS, SH** and **SJ**, *Disc* is evolutionary robust [46,47]. However, for cooperation to emerge, strategies and reputations must be coordinated: under **SH**, and despite the prevalence of the *Disc* strategy, defection still prevails over cooperation since individuals are mostly regarded as ***B***; **SS**, in turn, fails to prevent transitions into *AllD* in small populations; **SJ** fosters an ideal coordination between strategy and prevailing reputations, leading individuals to cooperate in the donation game.

The framework developed here has the advantage of being naturally extendable to social norms of higher order. Research carried out to date led to the discovery of **SJ** in a multi-level selection model in which an exhaustive search was carried out in the space of all third order norms [10,26]. Thus, it would not be surprising if **SJ** still promotes cooperation when this formalism is extended to third order norms. Work along these lines is in progress.

## Methods

### Actions conditioned to reputations

The actions employed in each interaction depend on the known reputation of the opponent. In a world of binary reputations (*Good*, ***G*** or *Bad*, ***B***), the strategy (also called action or behavioral rule) used by each player is a 2-bit string that prescribes an action (***C*** or ***D***) given the reputation of the opponent (***G*** or ***B***). Following the notation in [13–15], we denote a strategy by the duple *p* = (*p*_*G*_,*p*_*B*_), in which *p*_*G*_ and *p*_*B*_ represent, respectively, the probability of cooperating when the opponent is ***G*** or ***B***. There are thus 4 different strategies: (1,1), (1,0), (0,1) and (0,0) which are traditionally called *AllC*, *paradoxical Discrimination (pDisc)*, *Discrimination (Disc)*, and *AllD* [15].

We consider the existence of execution errors (*ε*) that simulate the inability of individuals to act in the way that their strategy dictates [11]. It is common practice to consider errors in the form of failed intended cooperation [15,31], due, for instance, the lack of *“resources*, *time or energy”* to donate [52]. Our results, however, remain valid even if the execution error would also induce defectors to involuntarily cooperate.

### Reputation dynamics

We assume that the donation game described in the main text is observed by a third party that will update the reputation of the players according to a social norm that is common to the entire population. The social norms prescribe a new reputation to a potential donor given the action employed (***C*** or ***D***) and the reputation of the opponent (the potential receiver of the donation). These second order social norms are defined as a bit-string with length 4, *d* = (*d*_*G*,*C*_,*d*_*G*,*D*_,*d*_*B*,*C*_,*d*_*B*,*D*_), in which *d*_*i*,*j*_ denotes the probability of assigning a *good* reputation to an individual that employed action *j* towards an opponent with reputation *i*.

There are 16 different second order social norms [15], which reduce to 10 if we take into consideration that the labels ***B*** and ***G*** can be swapped and the same results would ensue. In other words, norms *d*_1_ = (*d*_*G*,*C*_,*d*_*G*,*D*_,*d*_*B*,*C*_,*d*_*B*,*D*_) and *d*_2_ = (1-*d*_*B*,*C*_,1-*d*_*B*,*D*_,1-*d*_*G*,*C*_,1-*d*_*G*,*D*_) are equivalent due to a mirror symmetry [13].

We consider the existence of assignment errors, *α* [14]. They model the fact that the bystander observing the interaction may fail to attribute an accurate reputation to the donor, due to a myopic assess of the reputation of the potential receiver or due to a misinterpretation of the action employed. Following [10,13,14,22,23,26], and given that we are dealing with small communities, we assume that, once the reputation of an individual is assigned, it is widely and faithfully disseminated throughout the population, so that everyone shares the same opinion regarding the reputation of others.

### Update of reputations

In the **SMA**, we assume a maximum of two strategies (*p* and *p*') to be present, at any time, in the population. We assume that *p* already includes the execution error (i.e., *p*→(1−*ε*)*p*) and *d* already includes the assignment error (i.e., *d*→(1–2*α*)*d*+*α*). There are private errors, occurring with a probability *χ*, in assessing the actual reputation of an opponent. Consequently, denoting X = (1−*χ*,*χ*) and $\bar{Χ}$ = (*χ*,1−*χ*), the probability that someone using strategy *p* cooperates when meeting a *good* opponent is given by $C_{G}^{p}=(1-\chi )p_{G}+\chi p_{B}=X.p^{T}$, and the probability of cooperating with a *bad* opponent is given by $C_{B}^{p}=\chi p_{G}+(1-\chi )p_{B}=\bar{X}.p^{T}$. The probability that one observer assigns a *good* reputation to an individual using *p* and interacting with a *good* opponent is given by

$$G_{G}^{p}=(1-\chi )(C_{G}^{p}d_{G,C}+(1-C_{G}^{p})d_{G,D})+\chi (C_{G}^{p}d_{B,C}+(1-C_{G}^{p})d_{B,D})=(X⊗(C_{G}^{p},1-C_{G}^{p}))\cdot d^{T}$$

where ⊗ is the *Kronecker product* ((*a*_1_,*a*_2_)⊗(*b*_1_,*b*_2_) = (*a*_1_ *b*_1_,*a*_1_ *b*_2_,*a*_2_ *b*_1_,*a*_2_ *b*_2_)). The probability of assigning a *good* reputation to an individual using *p* and interacting with a *bad* opponent is given by $G_{B}^{p}=(\bar{X}⊗(C_{B}^{p},1-C_{B}^{p}))\cdot d^{T}$.

Given the expressions above, we now define birth and death probabilities [53] for *good* individuals. We use *h* and *h’* to denote the number of *good* individuals using strategies *p* and *p’*. For a population with size *Z*, where *k* individuals use strategy *p* (and *Z-k* use *p’*), the probability of having one more *good* individual using strategy *p* is given by,

$$H_{p}^{+}(h,h\prime )=\frac{k-h}{Z}(\frac{h+h\prime }{Z-1}G_{G}^{p}+\frac{Z-h-h\prime -1}{Z-1}G_{B}^{p})$$

whereas the probability of having one more *bad* individual using strategy *p* is given by,

$$H_{p}^{-}(h,h\prime )=\frac{h}{Z}(\frac{h+h\prime -1}{Z-1}(1-G_{G}^{p})+\frac{Z-h-h\prime }{Z-1}(1-G_{B}^{p}))$$

with analogous expressions for the birth and death probabilities associated with *good* individuals using the strategy *p’* (i.e. the expressions $H_{p^{\prime }}^{+}$ and $H_{p^{\prime }}^{-}$). To that end, one only has to substitute *k* for *Z-k*, *h* for *h’* and *p* for *p’*.

For a fixed value of *k*, the expressions $H_{p}^{+}$,$H_{p}^{-}$,$H_{p^{\prime }}^{+}$,$H_{p^{\prime }}^{-}$ define the stochastic process with which we may evolve the reputation dynamics in the population. Indeed, those probabilities define a two-dimensional Markov chain whose states, (*h*,*h’*), are defined by the number of *good* individuals using strategies *p* and *p’*. In total, there are *S = (k+1)(Z-k+1)* different states. The entry (*i*,*j*) of the underlying transition matrix (*H*) represents the transition probability from state $\left(h_{i},h\prime_{i}\right)$ to state $\left(h_{j},h\prime_{j}\right)$. Consequently, the entries of matrix *H* are given by

$$H_{i,j}\{\begin{array}{c} \begin{array}{c} H_{p}^{+}(h_{i},h\prime_{i}),\;h_{j}=h_{i}+1\wedge h\prime_{j}=h\prime_{i}\\ H_{p}^{-}(h_{i},h\prime_{i}),\;h_{j}=h_{i}-1\wedge h\prime_{j}=h\prime_{i} \end{array}\\ H_{p^{\prime }}^{+}(h_{i},h\prime_{i}),\;h_{j}=h_{i}\wedge h\prime_{j}=h\prime_{i}+1\\ H_{p^{\prime }}^{-}(h_{i},h\prime_{i})\text{, }h_{j}=h_{i}\wedge h\prime_{j}=h\prime_{i}-1\\ \begin{array}{c} H^{=}(h_{i},h\prime_{i}),\;i=j\\ 0,\text{ otherwhise} \end{array} \end{array}$$

where

$$H^{=}(h_{i},h\prime_{i})=1-H_{p}^{+}(h_{i},h\prime_{i})-H_{p}^{-}(h_{i},h\prime_{i})-H_{p^{\prime }}^{+}(h_{i},h\prime_{i})-H_{p^{\prime }}^{-}(h_{i},h\prime_{i})$$

is the probability of keeping the same reputation distribution.

From *H*, one can now compute the stationary (or limiting) distribution *σ*, defined as the eigenvector of matrix *H*, associated with eigenvalue 1 [54],

σH=σ

### Update of strategies

The evolution of strategies in the population is determined by a birth death process with imitation [55], in which those strategies that fare better are imitated more often [56,57]. This probabilistic imitation (i.e., the probability of strategy *p* being imitated by an individual previously adopting *p*', *P*(*p*'→*p*) is accomplished through the Fermi (also known as pairwise comparison) update rule [55,58], $P(p\prime \to p)=1/(1+e^{-\beta \;\Delta f_{p,p\prime }})$, where $\Delta f_{p,p\prime }(k)={\bar{f}}_{p}(k)-{\bar{f}}_{p\prime }(k)$ is the difference of average fitness between *p* and *p’* and *β* controls the selection strength: whenever *β*→0 imitation approximates the neutral drift; on the other hand, whenever *β*→+∞ the imitation occurs deterministically and selection pressure is maximal. To this end we compute the average payoff (fitness) of individuals employing a given strategy in the following way: The frequency-dependent fitness of strategy *p*, when *k* individuals are using it (and thereby *Z-k* are using *p’*), is composed by two terms: one positive corresponding to the received benefit (*b*), and another negative that translates the donations made (*c*) when individuals using *p* cooperate: *f*_*p*_(*k*,*h*,*h*') = *bR*_*p*_(*h*,*h*')−*cD*_*p*_(*h*,*h*'). *R*_*p*_ *(h*,*h’)* stands as the probability that a *p* strategist receives a donation,

$$R_{p}(h,h\prime )=\frac{h}{k}(\frac{k-1}{Z-1}C_{G}^{p}+\frac{Z-k}{Z-1}C_{G}^{p^{\prime }})+\frac{k-h}{k}(\frac{k-1}{Z-1}C_{B}^{p}+\frac{Z-k}{Z-1}C_{B}^{p^{\prime }})$$

*D*_*p*_ *(h*,*h’)*, in turn, stands as the probability that a *p* donates,

$$D_{p}(h,h\prime )=\frac{h}{k}(\frac{h-1+h\prime }{Z-1}C_{G}^{p}+\frac{Z-h-h\prime }{Z-1}C_{B}^{p})+\frac{k-h}{k}(\frac{h+h\prime }{Z-1}C_{G}^{p}+\frac{Z-h-1-h\prime }{Z-1}C_{B}^{p})$$

Provided a distribution of reputations *σ* is known, the average fitness is then calculated as ${\bar{f}}_{p}(k)=\sum_{0<h<k}\sum_{0<h\prime <Z-k}\sigma_{h,h\prime }f_{p}(k,h,h\prime )$, where *σ*_*h*,*h'*_ = *σ*_h(Z−k+1)+h')_ is the stationary distribution over the state in which there are *h* and *h’* individuals labeled ***G*** and using, respectively, action rules *p* and *p’*.

### Small-mutation approximation (SMA)

The fixation probability (*ρ*_*p*'→*p*_) of a unique mutant *p* in a population where *Z-1* individuals use *p’* can be written [4,53,55,59],

$$\rho_{p\prime \to p}={\left(1+\sum_{i=1}^{Z-1}\prod_{j=1}^{i}\frac{T^{-}(j)}{T^{+}(j)}\right)}^{-1}$$

Using the pairwise comparison rule (introduced above) to model the probability of imitation [55] this expression simplifies to

$$\rho_{p\prime \to p}={\left(1+\sum_{i=1}^{Z-1}\prod_{j=1}^{i}e^{-\beta \;\Delta f_{p,p\prime }}\right)}^{-1}$$

With these definitions for the fixation probabilities, we setup now an embedded Markov chain whose state-space is composed by all the possible monomorphic states,

$$T_{i,j}=\frac{\rho_{i\to j}}{3}(i\neq j)\;T_{i,i}=1-\sum_{k=1,k\neq i}^{3}\frac{\rho_{i\to k}}{3}$$

Following a procedure similar to that employed in the derivation of the stationary distribution of reputations, the stationary distribution of strategies is unique to the extent that the underlying Markov chain is irreducible, and given again by the eigenvector associated with the eigenvalue 1 of the transition matrix [41,45,54]).

### Cooperation index

The *cooperation index* (η) is computed, for a given social norm, by taking the weighted average of the fraction of cooperative acts that take place in each of the monomorphic configurations of the population; for weights, we use the fraction of time the population spends in each of these configurations, provided by the stationary distribution of strategies. Denoting by *λ*_*pi*_ the fraction of time spent in the monomorphic configuration where all individuals adopt *p*_*i*_, and denoting by *σ* ^*d*^(*p*_*i*_,*h*) the probability of having *h good* individuals within the monomorphic configuration *p*_*i*_ (calculated with *d* as the underlying social norm), the *cooperation index* (η) is given by

$$\eta =\sum_{p_{i}\in \{AllC,AllD,Disc,pDisc\}}^{}\lambda_{p_{i}}\sum_{j=0}^{Z}D_{p_{i}}(j,0)\sigma^{d}(p_{i},j)$$

## Supporting Information

S1 Text Supporting text. Supporting information containing 1 additional table (Table A) with the numerical data depicted in Fig 3 and 4 additional Figures A, B, C and D explaining and reporting the results of computer simulations in which the full-state space and high mutation rates are considered. Details are provided regarding the effect of population size for different mutation rates and different error rates. The distribution over the full-state space is detailed, resorting to the representation of a simplex together with the most prevalent states.
(PDF)
