# Supplementary material for: Social Norms of Cooperation in Small-Scale Societies
Source: PLoS Comput Biol. 2016 Jan 25;12(1):e1004709. doi: 10.1371/journal.pcbi.1004709 (PMC4726523; doi:10.1371/journal.pcbi.1004709)
Supplement: S1 Text — Supporting information containing 1 additional table (Table A) with the numerical data depicted in Fig 3 and 4 additional Figures A, B, C and D explaining and reporting the results of computer simulations in which the full-state space and high mutation rates are considered. Details are provided regarding the effect of population size for different mutation rates and different error rates. The distribution over the full-state space is detailed, resorting to the representation of a simplex together with the most prevalent states. (PDF) [file pcbi.1004709.s001.pdf]

(S1 Text) Supporting Information

Social norms of cooperation in small-scale societies

Fernando P. Santos[a,b], Francisco C. Santos[a,b], and Jorge M. Pacheco[c,d,b §]

[a] INESC-ID and Instituto Superior Técnico, Universidade de Lisboa, IST-Tagusparque, 2744-016 Porto Salvo, Portugal

[b] ATP-Group, P-1649-003 Lisboa Codex, Portugal

[c] Centro de Biologia Molecular e Ambiental, Universidade do Minho, 4710 - 057 Braga, Portugal

[d] Departamento de Matemática e Aplicações, Universidade do Minho, 4710 - 057 Braga, Portugal

Introduction

In this supplementary text we resort to large-scale computer simulations to confirm the surprisingly wide interval of parameters in which the analytical approximation employed in the main text is valid. The small mutation approximation (SMA) allows us to employ the analytical study presented in the main text, by confining the number of strategies co-existing in the population to a maximum of two. However, in general populations may exhibit, simultaneously, the presence of more than two strategies (particularly for higher exploration rates). Thus, it is important to verify to which extent this approximation remains useful whenever exploration rates increase. Here we confirm that even considering high exploration rates and a state space that allows for the co-existence of any combination of the four strategies *i)* only some social norms are able to promote cooperation in finite populations *ii)* form these, stern-judging and simple standing stand out as the *leading-two* and *iii)* stern-judging stands out as the one that promotes most cooperation in small populations. These numerical results also provide a sharper intuition on the dynamics occurring in the complete state space, in going beyond the analysis of transitions among monomorphic states.

Details of Computer Simulations

In the computer simulations, evolution proceeds in discrete steps involving imitation and mutation. At the beginning of one simulation (also called run), each individual adopts one of the 4 possible strategies, ((1,1), (1,0), (0,1), (0,0)), chosen using a Uniform Probability Distribution (UPD). Moreover, individual reputation (Good, G, or Bad, B) is also assigned with UPD. Each simulation is executed for a large number g of generations. In each generation, Z individuals chosen with UPD are given the opportunity of revising their strategy. After selecting one (individual A, with UPD) out of the Z individuals, strategy revision may happen

through *mutation* or *imitation*. Mutation happens with probability μ: a new strategy ((1,1), (1,0), (0,1), (0,0)) is randomly adopted (UPD). Imitation happens with probability $1-\mu$: a new individual is randomly selected (model — B, UPD), and the opportunity is given for A to update her/his strategy. The fitness of both individuals (F_A and F_B) is calculated as the average payoff earned in g=2Z games played against individuals in the population selected randomly with UPD. That large number of games is adequate to have a clear assessment of the average payoff, given the frequency of strategies present in the population, and to account for the dynamic reputation assignment described below. With probability ε the action employed by A is the opposite of the one dictated by her/his action rule (strategy), which mimics the execution error discussed in the main text. After each game, with a probability τ, the reputation of each individual is updated following the governing social norm. τ allows us to test different time-scales of reputation dynamics by controlling the average number of reputation updates per strategy update. Strictly, the analytical setup is recovered for $g\tau \to \infty$, yet, as illustrated below for various intermediate time-scales, the analytical results in the main text remain robust for a wide range of reputation assignment time-scales. If there is a reputation update, with probability α the assigned reputation is the opposite of the one dictated by the social norm, which is the analogous of the assignment error discussed in the main text. The information regarding the assigned reputation will spread over the entire population, i.e., reputations are public and implemented in the form of a vector available to the whole population. This is an approximation in at least two ways. First, information does not spread instantaneously. Second, information spreading is not error-free. Arguably, in small-scale societies the problem of the speed of information is less severe. Nonetheless, besides assuming different reputation assignment time-scales, below we also take into consideration the fact that reputation spreading is not error-free. Thus, each time an individual needs to assess a peer (either to decide about what action to employ or to decide what new reputation to attribute), the retrieved reputation

may be the opposite of the actual reputation of the opponent, with a probability (which is called private assessment error [15]) χ. Furthermore, individual A will acquire the strategy of B with a probability given by $(1+e^{-\beta(F_A-F_B)})^{-1}$ — the so-called Fermi update rule [55] — where β controls the selection strength in the way described before. We refer to F_A (and F_B) for notational simplicity: that quantity has a direct connection with f_p, discussed in the main text, as individual A (B) is acting with a strategy *p*, however, f_p is fully defined given *k*, *h*, and *h'*, while F_A depends on the specific strategy and reputation of the opponents selected to play with A. In Figure D we provide a pseudo-code illustrating the details of the computational method just described.

Results

We run our simulations R=100 different times, each one starting from a random setup. As stated below, series of *Z* — the population size — steps of imitation/mutation defines one generation. Each simulation lasts G=3×10^5 generations. The average cooperation index corresponds to the fraction of donations that occurred after a transient period of 0.1*G* generations. The average extends to the ensemble *R* of all simulations carried out. The cooperation index is computed by counting explicitly the number of donations and normalizing it by the total number of interactions.

Figure A shows the average cooperation index obtained analytically and numerically, for various population sizes and mutation rates, for the same social norms discussed in the main text. The numerical results assume two distinct intermediate time-scales of reputation assignment, which significantly differ from the limit adopted in the analytical description. **SJ** and, to a less extent, **SS** stand out for different orders of magnitude of the mutation rate. We therefore confirm that the results obtained in the main text remain qualitatively valid for a broad range of mutation rates and reputation assignment time-scales.

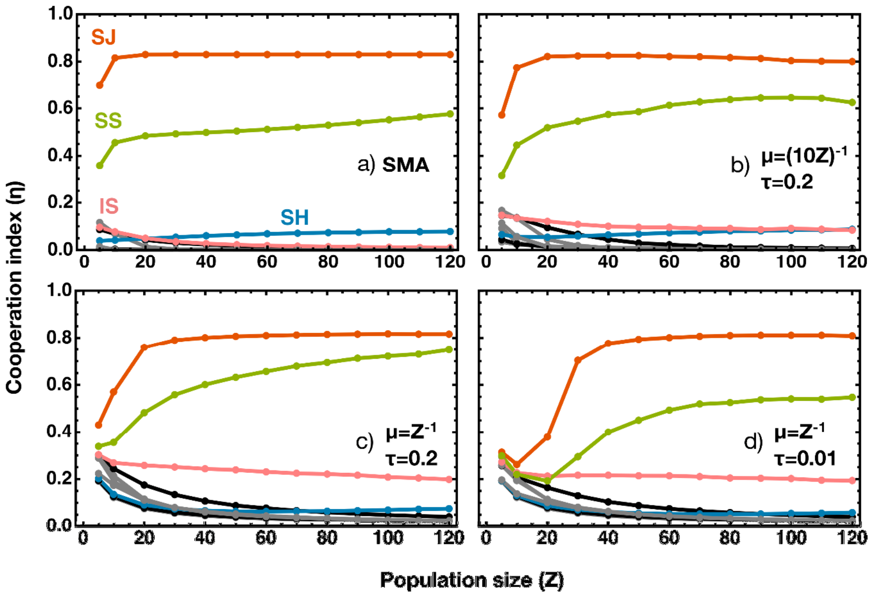

Figure A Cooperation index under the influence of different social norms and mutation rates for high execution errors. Panel a) reproduces the analytical results (**SMA**) already presented in Fig. 1 of the main text (used here as reference). In **Panel b)** we show the average cooperation index obtained from numerical simulations for the same parameters, assuming a mutation rate $\mu=(10Z)^{-1}$ and a value $\tau=0.2$ for the probability of reputation revision as a result of each interaction. In **Panels c)** and **d)** we show the cooperation index, respectively, considering a larger μ and lower τ, which imply further deviations from the assumptions made in the analytical model. The cooperation levels in both **SS** and **IS** profit from the increased noise introduced by higher mutation rates. Yet, the conclusions obtained from the analytical model remain valid for a broad range of mutation rates and intermediate time-scales of reputation revision, i.e., we confirm the advantage of **SJ** in small population sizes and the smooth convergence of the levels of cooperation obtained from **SS** and **SJ** for large population sizes. In particular, **Panels c)** and **d)** illustrate the validity of the analytical conclusions for a mutation rate much higher than the usual limit of validity for the SMA [S1]. The results regarding other norms [(B,B,B,B), (B,B,G,B). (B,B,G,G), (B,G,B,B), (B,G,G,B) and (G,G,B,B)] are colored with either gray or black; most lead to index values close to 0. Other parameters: b=5, c=1, $\alpha=\chi=0.01$, $\varepsilon=0.08$, and $\beta=1$.

It also worth pointing out that, while the prevalence of cooperation under **SJ** remains unaltered in the absence of a significant fraction of erroneus actions and assessments, other social norms can be signifcantly influenced. This is illustrated in Figure B, both analytically and numerically, where we pick the specific case of low execution errors (see Fig. 2 for a general picture of the impact of other types of errors). As an example, a low execution error hinders cooperation under **SS** and this effect is even more evident if the population size increases. If the execution error is low, *AllC* and *Disc* become almost neutral, opening a route for *AllD* to thrive. This effect becomes more evident as stochasticity is weakened by increasing the population size. This way, the prevalence around the monormorphic configuration *AllD* (relative to the prevalence in *Disc*) increases for large populations. Again, also in this case, **SJ**

shows a remarkable robustness, even assuming relatively slow time-scales of reputation revision (small values of τ).

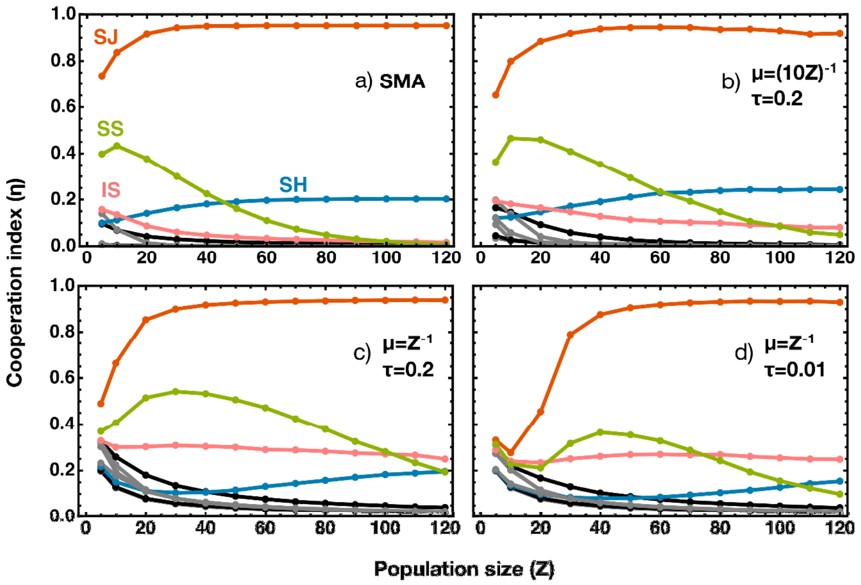

Figure B Cooperation index under the influence of different social norms and mutation rates for low execution errors. The results obtained employing the analytical framework (SMA, **Panel a)**) remain valid under high mutation rates (μ=$(10Z)^{-1}$ in **Panel b)** and μ=Z^{-1} in **Panels c)** and **d)**) and also for different time-scales of reputation assignment (0.2 in Panels **b)** and **c)** and 0.01 in Panel **d)**). Indeed, even considering high μ and low τ, i.e., departing from the assumptions made in **SMA**, the analytical framework is useful in describing the population size effects on the cooperation indexes supported by each social norm. In particular, combining low execution errors with large population sizes has a pernicious effect on the cooperation levels under **SS**, but has a limited impact on the performance of **SJ**. The results regarding other norms [(B,B,B,B), (B,B,G,B). (B,B,G,G), (B,G,B,B), (B,G,G,B) and (G,G,B,B)] are colored with either gray or black; most lead to index values close to 0. Other parameters: b=5, c=1, α=χ=ε=0.01, and β=1.

Finally, in Figure C we provide a representation of the most prevalent states — computed numerically for the leading norms **SJ** and **SS** and a mutation rate of μ=1/Z — in the entire state-space of configurations in which an arbitrary number of individuals may adopt each of the four different action rules. Each small sphere of the simplex represents a configuration in which the population spends a fraction of time higher than $1/Z^2$. The color gradient shows the prevalence of *Good* (**G**) and *Bad* (**B**) reputations in those states. Figure C allows us to conclude that Fig. 3 in main text provides an accurate intuition for the dynamics occurring in the full state-space, regarding the adoption of action rules and the reputation distribution (numerical values for the data represented in Fig. 3 are provided in Table A). Indeed, as Fig. 3 also depicts, for **SJ** most time is spent near the highly cooperative monomorphic states ALL-*Disc*

and ALL-*pDisc*, in which everyone is regarded, respectively, as ***G*** and ***B***. **SS**, in turn, is able to maintain a high prevalence near the configuration ALL-*Disc*: yet, it is weaker in preventing the fixation of *AllC* individuals (depicted in Figure C by the high prevalence in the edge *Disc-AllC*) and the consequent invasion of *AllD* (shown by the high prevalence near the monomorphic configuration *AllD*).

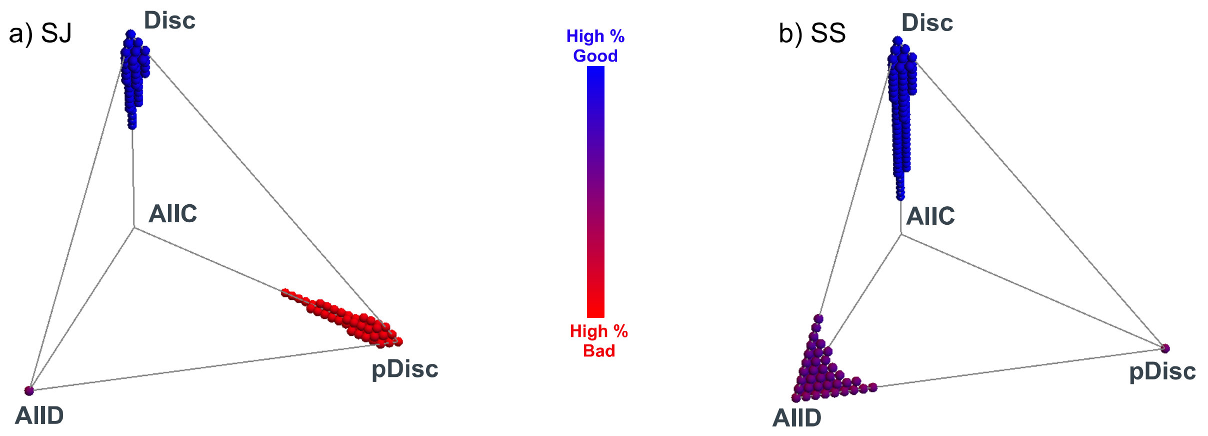

Figure C Prevalence of action rules and reputation distribution in the full state space. The full state space, pictured in three dimensions by a regular tetrahedron: each vertex represents a monomorphic configuration, in which all the population adopts the corresponding action rule; each edge contains those configurations in which at most two actions rules are adopted in the population and, following the same reasoning, faces contain the configurations in which three action rules are adopted in the population. We place a small sphere in the most prevalent states, here defined as configurations where more than $1/Z^2$ of the time is spent. The color gradient represents the distribution of reputations in that configuration. The left panel, a), shows the prevalence of each configuration when the ruling social norm is the Stern-Judging (**SJ**). The right panel, b), shows the same for Simple Standing (**SS**). Other parameters are: b=5, c=1, α=χ=ε=0.01, β=1, Z=40, μ=1/Z and τ=1.

	**ALLD**			**PDISC**			**DISC**			**ALLC**		
	%Coop	%Time	%Good	***%Coop***	***%Time***	***%Good***	%Coop	%Time	%Good	***%Coop***	***%Time***	***%Good***
**SH**	0	0.19	0.01	***0.9***	***0***	***0.02***	0.08	0.81	0.09	***0.92***	***0***	***0.16***
**SJ**	0	0	0.5	***0.83***	***0.5***	***0.1***	0.83	0.5	0.9	***0.92***	***0***	***0.5***
**IS**	0	0.95	0.01	***0.48***	***0.02***	***0.5***	0.15	0.02	0.16	***0.92***	***0.01***	***0.91***
**SS**	0	0.40	0.5	***0.46***	***0***	***0.5***	0.84	0.6	0.91	***0.92***	***0***	***0.92***

Table A Numerical data used to draw Figure 3 (main text). The average cooperation (%Coop) in each monomorphic configuration, together with the fraction of time (%Time) spent in that same configuration, provide the basis to calculate the cooperation index (η) depicted in Fig. 3 (main text). The average cooperation depends on the characteristics of the action rule fully adopted in the given monomorphic configuration, on the social norm that dictates the reputation assignment and the consequent fraction of individuals with G label (%Good) and B label (1-%Good). Parameters (as in Fig. 3, main text): Z=50, b=5, c=1, α=χ=0.01, ε=0.08, β=1.

Runs: number of runs;
Gens: number of generations;
Z population size;
P: vector of all individual strategies;
P_k: strategy of individual k;
D: vector of all individual public reputations;
D_k: public reputation of individual k;
$\mathcal{U}\{a,b\}$: uniform distribution over integers between a and b;
$Rand \sim \mathcal{U}(0,1)$: random value sampled following the standard uniform distribution;
μ : mutation probability;
ϵ : execution error probability;
α : reputation assignment error probability;
χ : private assessment error probability;
τ : reputation update probability;
F_a : fitness of individual a;
$d(C_x, D_y)$: new reputation given social norm d, action C_x and reputation D_y;
$\Pi(x,y)$: payoff to individual x after an interaction with y where both x and y may donate following their strategies (P_x and P_y) and reputations (D_x and D_y). An update of reputations occurs. This step takes into account the execution, assignment and assessment errors.
AllD $\equiv 0$; **pDisc** $\equiv 1$; **Disc** $\equiv 2$; **AllC** $\equiv 3$;
Cooperate $\equiv 1$; **Defect** $\equiv 0$;
Good $\equiv 1$; **Bad** $\equiv 0$;

```
for r ← 1 to Runs do
    for k ← 1 to Z do
        P_k ← X ~ U{0,3}
        D_k ← X ~ U{0,1}
    end
    for t ← 1 to Gens do
        a ← X ~ U{1,Z}
        if Rand < μ then P_a ← X ~ U{0,3};
        else
        b ← X ~ U{1,Z}, b ≠ a;
        F_a ← 0;
        F_b ← 0;
        for i ← 1 to 2Z do
            c ← X ~ U{1,Z}, c ≠ a;
            F_a ← F_a + Π(a,c);
            /* update D_a and D_c */
            c ← X ~ U{1,Z}, c ≠ b;
            F_b ← F_b + Π(b,c);
            /* update D_b and D_c */
        end
        F_a ← F_a/(2Z)
        F_b ← F_b/(2Z)
        if Rand < (1 + e^(F_a − F_b))^(−1) then P_a ← P_b;
         /* keep track of the average number
        of cooperations */;
    end
end
```

```
Π(x,y) :
if Rand < χ then
    if (Rand < ε) ∧ (P_x(1 − D_y) == 1) then
        C_x ← 1 − P_x(1 − D_y);
    else
        C_x ← P_x(1 − D_y);
    end
else
    if (Rand < ε) ∧ (P_x(D_y) == 1) then
        C_x ← 1 − P_x(D_y);
    else
        C_x ← P_x(D_y);
    end
end
if Rand < τ then
    if Rand < α then
        D_x ← 1 − d(C_x, D_y);
    else
        D_x ← d(C_x, D_y);
    end
end
/* D_y is updated similarly to D_x */
/* C_y, which is 1 if y cooperates with
x and 0 otherwise, is calculated
similarly to C_x */
return b.C_y − c.C_x;
```

Figure D Pseudo-code illustrating the computational method.

References

S1. Traulsen A, Hauert C, De Silva H, Nowak MA, Sigmund K (2009) Exploration dynamics in evolutionary games. Proc. Natl. Acad. Sci. USA 106: 709-712.
